# Analysis of HLA genotype in the recipients’ different tissues after haploidentical hematopoietic stem cell transplantation

**DOI:** 10.3389/fimmu.2026.1725621

**Published:** 2026-02-25

**Authors:** Jie Liu, Bing-Na Yang, Zhan-Rou Quan, Yin-Ming Zhang, Jia-Min Song, Zhi-Hui Deng, Hong-Yan Zou

**Affiliations:** Institute of Transfusion Medicine, Shenzhen Blood Center, Shenzhen, Guangdong, China

**Keywords:** buccal swab, chimerism, haplo-HSCT, HLA, loss of heterozygosity, peripheral blood, saliva

## Abstract

**Introduction:**

Peripheral blood samples are widely used in HLA genotyping due to their easy accessibility and the high-quality DNA from nucleated leukocytes. However, in cases of disease relapse requiring a second transplantation, clinicians encounter significant challenges in performing HLA genotyping and interpreting complex results from patients who have undergone haploidentical hematopoietic stem cell transplantation (haplo-HSCT). Furthermore, systematic studies investigating the impact of haplo-HSCT on recipients’ HLA genotypes across different tissues remain scarce. Therefore, this study aims to analyze HLA genotypes in various tissues of the recipient after haplo-HSCT.

**Methods:**

A total of 66 patients who received haplo-HSCT were enrolled, with peripheral blood, buccal swab and saliva samples collected for HLA genotyping. The results were compared with pre-HSCT HLA genotypes of the patients and their respective donors.

**Results:**

The majority of peripheral blood samples (55/57) exhibited the donor’s HLA genotype, whereas most buccal swabs (54/62) retained the patient’s pre-HSCT genotype. Among 20 salivary samples, 45% of patients retained their pre-HSCT genotype, while 30% exhibited the donor’s genotype. Notably, chimerism and HLA loss were detected in buccal swab and saliva cells of certain recipients. More strikingly, one patient’s buccal sample displayed complete donor HLA genotype replacement.

**Conclusion:**

These findings enhance our comprehension of the genetic effects of haplo-HSCT on the recipient’s different tissues and provide valuable insights for the rational selection of tissue samples and data interpretation in HLA genotyping for patients who underwent allogenic HSCT.

## 1 Introduction

Hematopoietic stem cell transplantation (HSCT) is a promising strategy for the treatment of multiple blood malignancies, bone marrow failure syndrome or genetic diseases (1, 2). HLA located in the short arm of chromosome 6, is the most complex genetic polymorphism system and has important clinical significance in organ or hematopoietic stem cell transplantation (3). The matching status of HLA between the donor and recipient is highly related to the outcomes of HSCT. In principle, it is preferential to select donors with fully matched HLA to avoid the occurrence of graft-versus-host disease (GVHD) and improve clinical outcome (4, 5). However, haploidentical HSCT (haplo-HSCT), wherein the donor shares only one HLA haplotype with the recipient, has emerged as a feasible and efficient therapeutic option for patients when they can’t find a fully HLA-matched donor with the advancement of immunomodulatory medical technology and the development of T-cell depletion techniques (6).

For a long time, it was believed that complete donor hematopoiesis is necessary to maintain engraftment after allogenic HSCT. A few decades ago, it was widely understood that the hematopoiesis of donor and recipient may coexist and this state of coexistence of hematopoietic cells is called mixed chimerism (7). Stable mixed chimerism is usually not associated with poor outcomes in non-malignant diseases such as aplastic anemia when donor-derived progenitor cells produce a sufficient number of erythrocytes and recipients achieve effective hematopoiesis with transfusion independence (8). However, recipient chimerism in patients with hematologic malignancies may foretell disease relapse. Therefore, assessment of chimerism was carried out monthly after transplantation to monitor a reappearance of the host hematopoiesis and guide the appropriate interventions such as immunosuppression regimen, donor lymphocyte infusion, and/or salvage second transplantation to treat the residual malignant cells (9, 10).

HLA is the key molecule mediating immune recognition of “self” and “non-self” (11). Donor T-cell infusions following haplo-HSCT can enhance immune reconstitution and exert anti-leukemic effects by targeting mismatched HLA antigens (12, 13). However, in this strong immune stress state, homologous recombination may occur in the mismatched HLA gene region, which is replaced by the matched HLA genes, resulting in homozygosity, or loss of heterozygosity (LOH) (14, 15). LOH could occur in the entire HLA haploid region, or in some regions, such as HLA-class I and HLA-class II, as well as individual gene loci, which allowed tumor cells to escape the immune surveillance and often caused disease relapse (15). Loss of the mismatched HLA loci in leukemic patients after HSCT was numerously reported (16–18). In such patients, haploidentical donors with a new mismatched haplotype may improve outcomes after second HSCT for relapsed hematologic malignancies (19). Therefore, assessment of HLA loss allows transplant centers to make quick decisions on the most appropriate therapies and/or alternative donor selection for rescue HSCT (15).

In clinical testing, peripheral blood samples are commonly used for HLA genotyping because they are easily accessible and contain nucleated leukocytes, which provide high-quality DNA that can be extracted for accurate amplification and sequencing of HLA genes (20). However, for patients who have undergone haplo-HSCT, their peripheral blood typically exhibits the donor’s HLA genotype. In this case, it is necessary to collect other tissue samples for HLA genotyping to make a comprehensive assessment. In practice, buccal swab and saliva samples are often selected. However, rare studies have investigated the genomic difference in various tissues resulting from allogenic HSCT. Furthermore, there is a lack of relevant clinical standards to guide the rational selection of tissue samples used for HLA genotyping for the patients receiving allogeneic HSCT.

In order to investigate the effect of haplo-HSCT on HLA genotypes in the recipients’ various tissue cells, we retrospectively identified patients who have undergone a haplo-HSCT from their family donors and were proposed to receive a second HSCT due to disease progression or relapse. Their HLA genotyping results of different tissues including peripheral blood, buccal swabs and saliva were collated and compared with those of pre-HSCT patients and their corresponding donors. Our study revealed that after haplo-HSCT, the recipient’s peripheral blood typically exhibited the donor’s HLA genotype, whereas the buccal swabs mainly retained the patient’s pre-HSCT genotype. Salivary samples can display the donor’s genotype or the patient’s pre-HSCT genotype. Moreover, chimerism and HLA loss were also observed in the buccal swab or saliva samples of certain recipients in our study. Unexpectedly, one patient’s buccal swab was observed to display the complete donor HLA genotype. Although the underlying mechanisms of these phenomena remain elusive, these findings may prompt further consideration and discussion among peers, fostering continued exploration in this area.

## 2 Materials and methods

### 2.1 Sample collection

The study population consisted of patients who had previously undergone a haplo-HSCT and were being evaluated for a second HSCT due to relapse or progression in the past two years, which represented a selected subgroup rather than a general post-haplo-HSCT population. Their peripheral blood, buccal swabs or saliva were collected for DNA extraction and HLA genotyping. This study was approved by the Ethics Committee of Shenzhen Blood Center (SZBCMEC-2025-023) and informed consent was obtained from all participants.

### 2.2 Genomic DNA extraction

The peripheral blood samples were anti-coagulated by EDTA-K2. The buccal swabs were soaked in sterile water for 1 hour, then centrifuged at 4000rpm for 10 min. The supernatant was discarded and the precipitate was resuspended with 400 μL deionized water. The supernatant of the saliva samples was discarded and the remaining liquid was mixed evenly. Then 400 μL samples described above were used for the extraction of genomic DNAs with MagCore^@^ DNA extraction kit (RBC Bioscience Corp., New Taipei, Tai Wan). Concentration and purity of DNA samples were measured and adjusted with the NanoDrop 2000 spectrometer (Thermo scientific, MA, USA) to reach the optimal concentration of 10–100 ng/μL.

### 2.3 HLA genotyping with PCR-Flow-rSSO

DNA fragments in *HLA-A, HLA-B, HLA-C, HLA-DRB1* and *HLA-DQB1* loci were amplified, hybridized with magnetic beads linked by specific oligonucleotide, and labelled by fluorescent with the use of LABType™ rSSO HD reagent. After detected by Luminex FlexMap 3D multifunctional flow detector, the typing results were analyzed using HLA Fushion v4.2 software.

### 2.4 HLA genotyping with NGS

HLA genotyping was performed with the commercial HLA typing kit (AllType FASTPlex NGS 11 loci HLA typing kits, One Lambda Inc., USA) on the Illumina MiSeqDx™ platform (Illumina, Inc., San Diego, CA, USA) as described previously (21). Allele assignment was conducted through the TypeStream Visual 3.1 NGS Analysis software (One Lambda, Thermo Fisher Scientific, CA, USA) according to the IPD-IMGT/HLA Database.

### 2.5 Analytical criteria for chimerism and LOH

When rSSO analysis software provides no definitive result and suggests the presence of three alleles, along with the observation of high background signals at multiple HLA loci in the NGS data, these combined findings strongly indicate the presence of chimerism. To confirm and quantify chimerism, HLA genotypes of the patient before HSCT and the corresponding donor are compared and the donor-specific alleles are incorporated into the sequence alignment within the TypeStream Visual 3.1 software. By analyzing the heterozygous positions between the alleles assigned by the software and the donor-specific alleles, the read-percentage can be estimated. A minor allele fraction exceeding 10% is typically considered as evidence of chimerism.

LOH is suspected when both rSSO and NGS analyses yield homogeneous results, but the pre-transplant HLA genotypes of the patient and corresponding donor exhibit heterogeneity. LOH is confirmed if the genotyping results only contain the shared allele between the patient and donor, with complete absence of the mismatched allele that was originally present in the patient before HSCT. It is crucial to note that LOH can occur with varying scopes, encompassing the entire HLA haploid region, specific regions such as HLA - class I and HLA - class II, or even individual gene loci within the HLA complex.

## 3 Results

### 3.1 Patient cohort

The clinical characteristics of patients were shown in **Table 1**. A total of 66 patients (41 males and 25 females) receiving haplo-HSCT were enrolled in this study, including 32 patients (48.5%) with acute myeloid leukemia (AML), 27 patients (40.9%) with acute lymphoblastic leukemia (ALL), 6 patients (9.1%) with myelodysplastic syndrome (MDS) and 1 patient (1.5%) with aplastic anemia (AA). The median age of these patients at transplantation was 35 years old with a range from 5–66 years old. Donors were mainly from the haploidentical siblings (n=28, 42.4%), followed by the parents (n=20, 30.3%) and offspring (n=18, 27.3%) of the patients. These patients were recommended for a second transplantation as they experienced disease progression or relapse within 6~59 months after their initial haplo-HSCT. HLA genotyping was performed to facilitate the selection of a suitable new donor.

**Table 1:** The clinical characteristics of patients.

Patient characteristics	N (%)
Number of patients	66
Gender	
female	25 (37.9)
male	41 (62.1)
Age	
median years (range)	35 (5 y - 66 y)
Diseases	
AML	32 (48.5)
ALL	27 (40.9)
MDS	6 (9.1)
AA	1 (1.5)
Type of donor	
Parents	20 (30.3)
Siblings	28 (42.4)
Offspring	18 (27.3)

### 3.2 HLA genotype in recipients’ different tissues after haplo-HSCT

**Figure 1:**
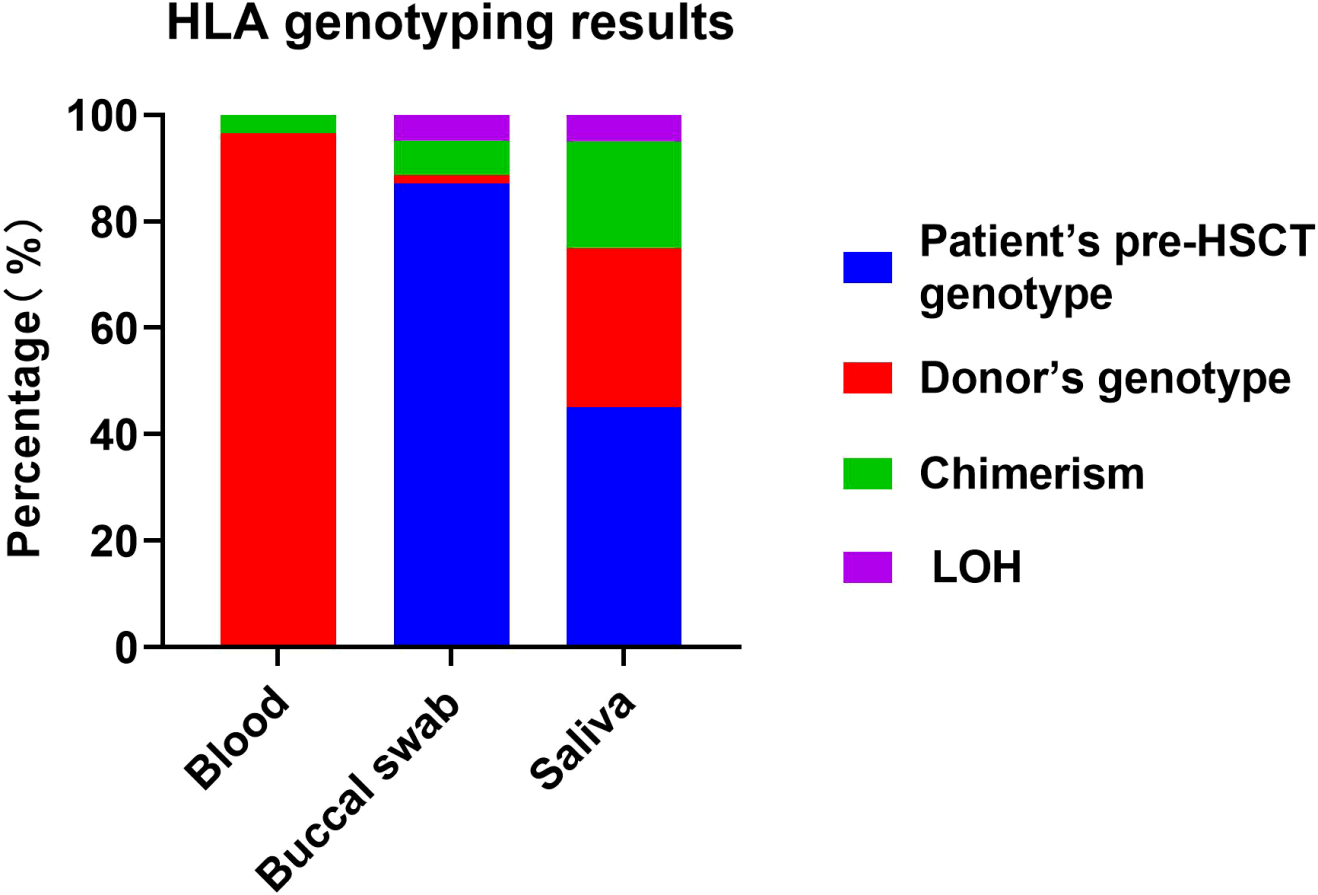
The distribution of HLA genotypes in the recipients’ different tissues after haplo-HSCT. Blue represented that the HLA genotyping result was consistent with the genotype of the patient before transplantation. Red represented that the HLA genotyping result was consistent with the donor’s genotype. Green represented that the HLA genotyping results exhibited chimerism. Purple represented that the HLA genotyping results exhibited loss of heterozygosity (LOH).

To investigate the impact of haplo-HSCT on the recipients’ HLA genotype in different tissues, the peripheral blood, buccal swab and saliva samples from the patients were collected for HLA genotyping. The results were then compared with the corresponding HLA genotypes of the patients prior to transplantation and those of their respective donors. The HLA genotyping results of all the investigated patients were provided in **Supplementary Table S1**. As summarized in **Table 2**, the majority of peripheral blood samples (55/57) displayed the donor’s HLA genotype, with only 2 cases showing chimerism. Conversely, the majority of buccal swabs (54/62) retained the patients’ pre-HSCT genotype, with one sample displaying the donor’s genotype, four showing chimerism, and three exhibiting LOH phenomenon. Furthermore, a total of 20 saliva samples were analyzed, among which 9 samples preserved the patient’s pre-HSCT genotype, six exhibited the donor’s genotype, four showed chimerism, and one presented LOH. The distribution of HLA genotype for each tissue type was depicted in **Figure 1**. The findings revealed that the peripheral blood is notably susceptible to the effects of haplo-HSCT, predominantly exhibiting the donor’s HLA genotype, indicating the successful engraftment and in harmony with the intended clinical purpose of HSCT. In contrast, buccal swab samples remain largely unaltered, generally retaining the patient’s original HLA genotype, which provides a robust rationale for selecting buccal cells as the optimal material for HLA genotyping in patients with a history of allogenic HSCT.

**Table 2:** HLA genotype in the recipients’ different tissues after haplo-HSCT.

Tissue types HLA genotype	Peripheral blood N (%)	Buccal swab N (%)	Saliva N (%)
Patient’s pre-HSCT genotype	0	54(87.1)	9(45.0)
Donor’s genotype	55(96.5)	1(1.6)	6(30.0)
Chimerism	2(3.5)	4(6.5)	4(20.0)
LOH	0	3(4.8)	1(5.0)
Total number	57	62	20

### 3.3 Chimerism of donor-specific HLA genes in the recipient’s buccal swab sample after haplo-HSCT

In the studied patient cohort, a 15-year-old patient (sample ID 17831) with ALL underwent haplo-HSCT with his mother as the donor in August 2023. The patient experienced a disease relapse in February 2024, prompting the proposal for a second transplantation. His peripheral blood and buccal swab samples were collected for HLA genotyping to facilitate selection of an unrelated HLA-matched donor, and the results were compared with the patient’s pre-HSCT genotype and his donor’s genotype.

**Figure 2:**
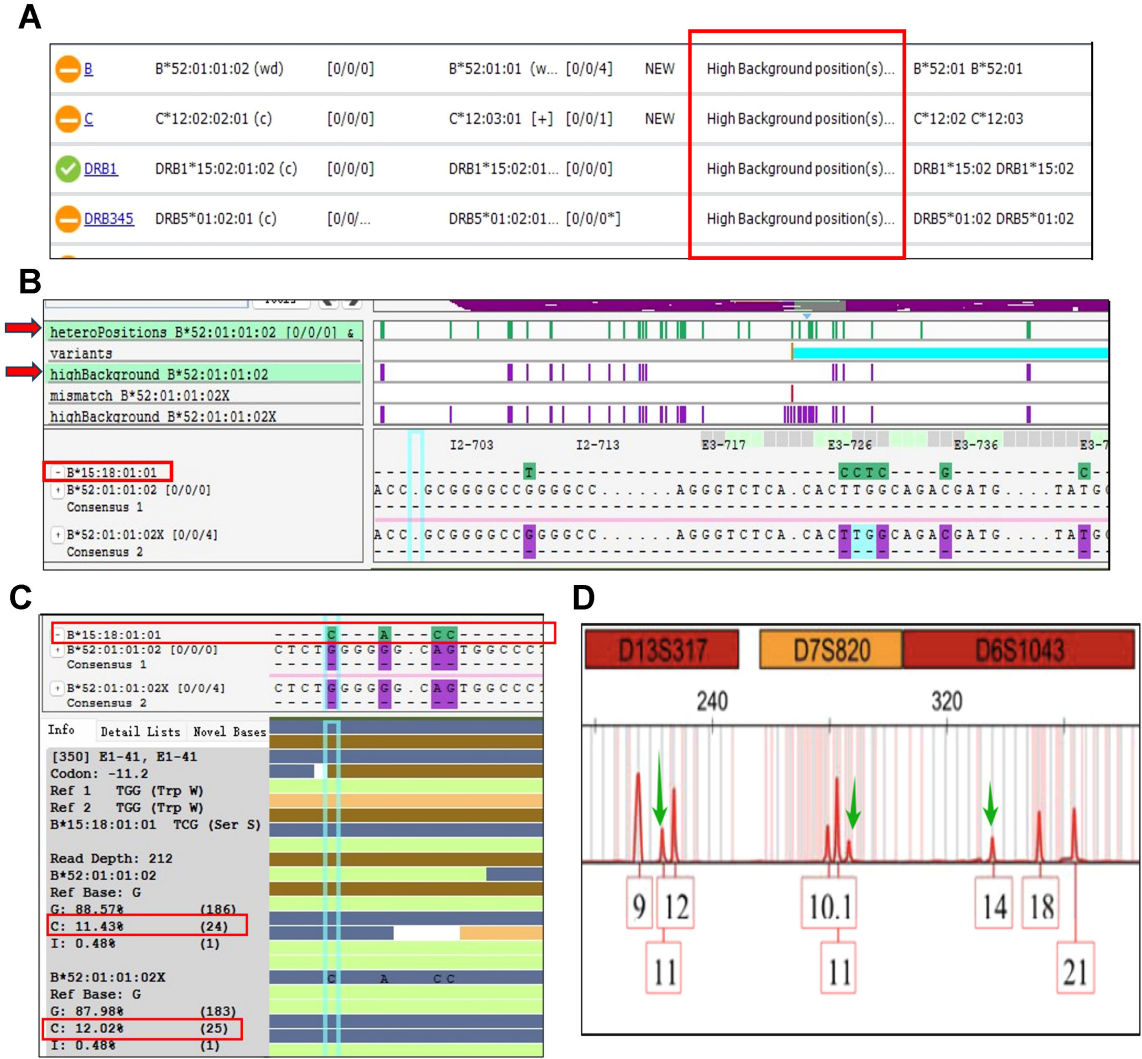
Chimerism of donor-specific HLA genes was observed in the recipient’s buccal swab after haplo-HSCT. **(A)** High background signals were observed at numerous HLA loci in the recipient’s HLA genotyping results. **(B)** The hetero-positions between B*52:01 and B*15:18 exhibited high background signals. Green showed the hetero-positions of B*52:01 and B*15:18. Purple showed the positions with high background signals indicating the existence of other nucleotides besides those of the assigned alleles. **(C)** The chimerism of B*52:01 and B*15:18 was observed in E1–41 position. **(D)** Chimerism of donor-recipient was observed in STR testing results. The green arrows indicated the existence of the third peak.

As shown in **Table 3**, the patient’s peripheral blood exhibited the donor’s HLA genotype, whereas the buccal swab generally retained his pre-HSCT genotype with about 11% chimerism of donor-specific HLA genes (as denoted in the brackets). In detail, firstly the TypeStream Visual 3.1 software revealed high background signals in exons of *HLA-B, C* and *DRB1* loci (**Figure 2A**). Focusing on the HLA-B locus as an illustrative example, despite its assignment as B*52:01, 52:01, when the donor-specific allele B*15:18 was incorporated into the sequence alignment, numerous heterozygous positions between B*52:01 and B*15:18 exhibited high background signals (**Figure 2B**). Specifically, at the E1–41 position (**Figure 2C**), 24 reads (11.43%) and 25 reads (12.02%) across the two alleles were sequenced as nucleotide C respectively, aligning with the B*15:18 allele. Similar conditions were also observed in other variant positions within the B*52:01/B*15:18. It was estimated approximately 11% of the reads at *HLA-B* locus corresponded to the B*15:18 allele. Chimerism of donor specific HLA genes was also detected in other HLA loci. Furthermore, the presence of donor gene chimerism was confirmed via short-tandem-repeat (STR) amplification testing, as evidenced by the detection of the third peaks, which were indicated by the green arrows (**Figure 2D**).

**Table 3:** Chimerism of donor genes in the recipient’s buccal swab samples after haplo-HSCT.

Loci	Patient before HSCT	Donor	Peripheral blood after HSCT	Buccal swab after HSCT
HLA-A	31:01, 33:03	11:01, 33:03	11:01, 33:03	31:01, 33:03(11:01)
HLA-B	52:01, 52:01	15:18, 52:01	15:18, 52:01	52:01, 52:01(15:18)
HLA-C	12:03, 12:02	07:04, 12:02	07:04, 12:02	12:03, 12:02(07:04)
HLA-DRB1	15:02, 15:02	15:01, 15:02	15:01, 15:02	15:02, 15:02(15:01)
HLA-DQB1	06:01, 06:01	06:02, 06:01	06:02, 06:01	06:01, 06:01(06:02)

Alleles in the brackets represented the donor-specific alleles detected at a low frequency.

These findings indicate that while buccal swabs generally retain the recipient’s original genotype after haplo-HSCT, instances of donor genetic chimerism do occur in certain cases. However, the underlying mechanism behind this phenomenon remains elusive. One possible explanation is that the donor-derived stem cells migrated into the buccal region and subsequently differentiated into epithelial cells, but further exploration is required to validate this hypothesis.

### 3.4 One recipient’s buccal swab totally exhibited the donor’s HLA genotype after haplo-HSCT

More unexpectedly than the occurrence of donor gene chimerism, we observed a rare phenomenon where the buccal swab of a patient (sample ID 16836) totally displayed the donor’s HLA genotype. This particular case involved a male patient suffering from ALL, who underwent haplo-HSCT from his daughter at the age of 42 in February 2021. Prior to the transplantation, HLA genotyping of the patient and his daughter revealed a 7/10 HLA matching status, as detailed in **Table 4**.

**Table 4:** One recipient’s buccal swab totally exhibited the donor’s HLA genotype after haplo-HSCT.

Loci	Patient before HSCT	Donor	Peripheral blood after HSCT	Buccal swab after HSCT
HLA-A	02:06, 11:01	02:07, 11:01	02:07, 11:01	02:07, 11:01
HLA-B	48:01, 15:46	46:01, 15:46	46:01, 15:46	46:01, 15:46
HLA-C	08:01, 03:03	01:02, 03:03	01:02, 03:03	01:02, 03:03
HLA-DRB1	15:01, 09:01	15:01, 09:01	15:01, 09:01	15:01, 09:01
HLA-DQB1	03:03, 06:02	03:03, 06:02	03:03, 06:02	03:03, 06:02

Two and a half years post-transplantation, the patient experienced a relapse of his disease. To aid in the selection of a new unrelated HLA-matched donor, HLA genotyping was performed on samples collected from the patient’s buccal swab and peripheral blood. Surprisingly, both the peripheral blood and buccal swab exhibited the donor’s HLA genotype. Typically, buccal cells retain the patient’s original genotype, and the detection of a donor genotype in recipient buccal swab samples is extremely rare, with the underlying mechanism remaining largely unexplained. Despite this, we have chosen to document this case to foster discussion and encourage deeper exploration among our peers.

### 3.5 Loss of heterozygosity of HLA genes in the recipient’s buccal swab and saliva after haplo-HSCT

HLA loss is commonly reported in leukemia cells, as these cells exploit this mechanism to evade immune detection, a process that has been linked to disease relapse. Interestingly, our study also identified HLA loss in non-hematopoietic tissues, including buccal and saliva samples. One AML patient (sample ID 18320) underwent haplo-HSCT with her son as the donor in December 2021 at the age of 44. The patient suffered the disease relapse 28 months after transplantation, prompting a recommendation for a rescue HSCT. Samples of her peripheral blood, buccal swabs and saliva were collected for HLA genotyping, and the results were subsequently compared with the HLA genotypes of the patient prior to HSCT and those of the donor, as illustrated in **Table 5**.

**Table 5:** HLA loss in the recipient’s buccal swab and saliva samples after haplo-HSCT.

Loci	Patient before HSCT	Donor	Peripheral blood after HSCT	Buccal swab and saliva after HSCT
HLA-A	30:01, 01:01	30:01, 24:02	30:01 (65%), 24:02 (35%)	30:01, 30:01
HLA-B	13:02, 15:17	13:02, 40:06	13:02 (75%), 40:06 (25%)	13:02, 13:02
HLA-C	06:02, 07:01	06:02, 08:01	06:02 (75%), 08:01 (25%)	06:02, 06:02
HLA-DRB1	07:01, 13:02	07:01, 12:01	07:01 (75%), 12:01 (25%)	07:01, 07:01
HLA-DQB1	02:02, 06:04	02:02, 03:01	02:02 (55%), 03:01 (45%)	02:02, 06:04

Rates in the brackets represented the proportion of corresponding allele.

**Figure 3:**
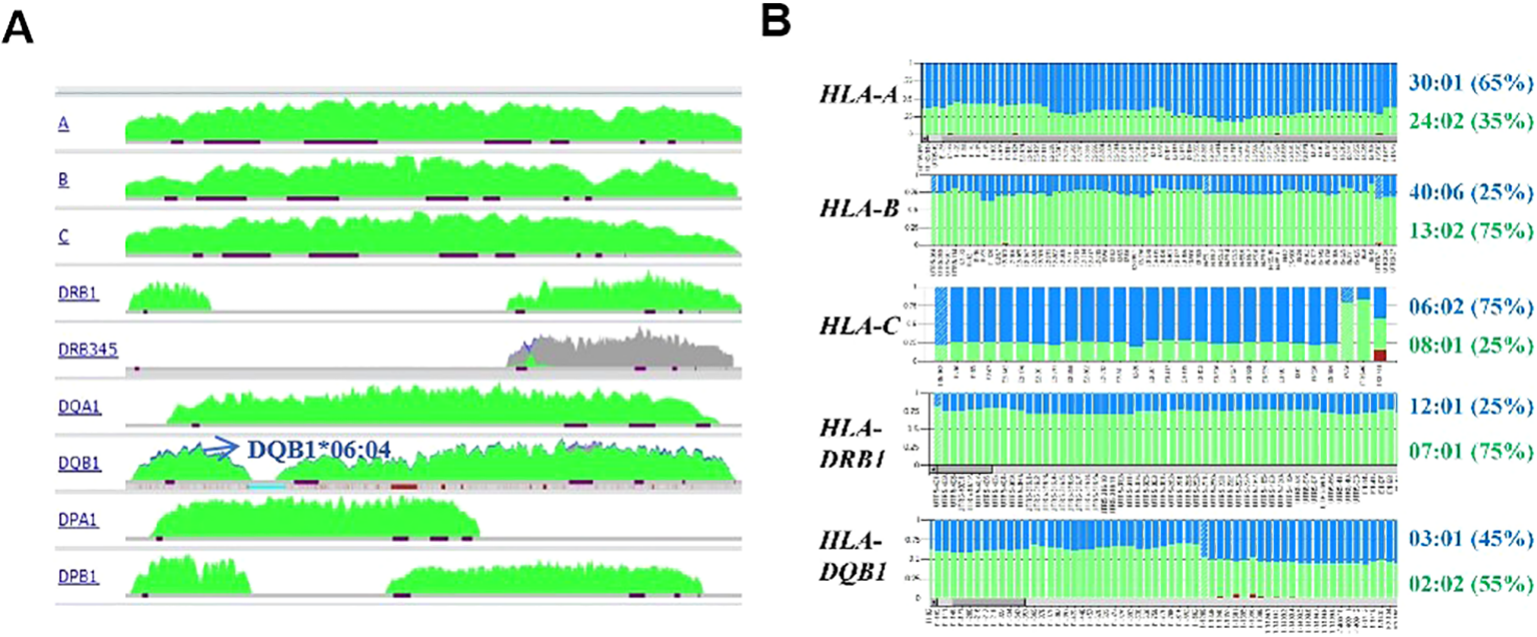
LOH and allelic imbalance were observed in the recipients’ HLA genotyping results after haplo-HSCT. **(A)** HLA genotyping revealed extensive homozygosity across multiple HLA loci in the recipient’s buccal swab and saliva samples. Allelic variants were color-coded for visualization: green denoted allele 1, blue denoted allele 2, and red horizontal lines specifically marked the exon regions of each HLA locus **(B)** Allelic imbalance was observed in multiple HLA loci in peripheral blood samples. The distribution of the varied nucleotides at heterozygous positions for the *HLA-A, B, C, DRB1* and *DQB1* loci were color-coded (green for allele 1, and blue for allele 2). The estimated percentages of each HLA allele were noted in the brackets.

It was found that in the patient’s buccal swab and saliva samples after haplo-HSCT, the recipient specific HLA genes A*01:01, B”15:17, C*07:01, DRB1*13:02, or the mismatched HLA alleles between the donor and pre-HSCT patient were lost. As depicted in **Figure 3A**, the TypeStream Visual 3.1 software demonstrated that numerous HLA loci exhibited homozygosity. Although the *DQB1* locus displayed two distinct alleles, the proportion of DQB1*06:04 was notably low. Collectively, above results demonstrated that in this patient, LOH occurred at the *HLA-A, B, C*, and *DRB1* loci, while allelic imbalance was evident at the *HLA-DQB1* locus. This result revealed that apart from leukemia cells which achieve immune evasion via HLA loss, the phenomenon of HLA loss can also occur in other tissues, including buccal swab and saliva samples. This discovery has been scarcely documented in existing literature, and the underlying mechanism warrants further in-depth investigation.

Regarding the peripheral blood sample, it predominantly exhibited the donor’s HLA genotype, but allelic imbalance was observed at the *HLA-A, B, C*, *DRB1* and *DQB1* loci. The proportions of variant nucleotides between the two alleles at each locus were detailed in **Figure 3B**, with the estimated percentages of the two alleles also listed in **Table 5**. Notably, the percentage of shared HLA alleles between the donor and recipient was higher compared to that of unmatched alleles. These findings may result from the coexistence of donor-derived blood cells and the relapsed leukemic cells exhibiting LOH. However, further investigation is required to validate this hypothesis.

## 4 Discussion

Haplo-HSCT has evolved as a promising option for patients with high-risk hematologic cancers when lacking an HLA-matched donor (22–24). However, with the recurrence of the disease and the subsequent recommendation for a second transplantation, clinicians face significant challenges in conducting HLA genotyping and interpreting the intricate results obtained from the recipient’s different samples. Moreover, there is a scarcity of systematic studies that thoroughly investigate HLA genotyping results in patients who have undergone haplo-HSCT or HLA-mismatched HSCT.

In order to analyze the influence of haplo-HSCT on HLA genotypes in the recipients’ different tissues, we specifically selected and enrolled patients who had previously undergone a haplo-HSCT and were being evaluated for a second HSCT due to relapse or progression in the past two years. These participants’ peripheral blood, buccal swabs and saliva were collected and subjected to HLA genotyping. Through comparison with the HLA genotypes of pre-HSCT patients and their corresponding donors, we found that peripheral blood samples generally exhibited the donor’s genotype, while the majority of buccal swabs displayed recipient’s pre-HSCT HLA genotype. And the saliva samples showed a mix of donor and recipient HLA genotypes. These disparities in HLA genotypes might be explained based on the cellular origins within these tissues. Peripheral blood predominately exhibited the donor’s genotype because recipients underwent marrow ablation prior to HSCT, and upon successful engraftment of the donor’s hematopoietic stem cells, these cells differentiated into various blood cell lineages, thereby reflecting the donor’s genetic profile. In contrast, the primary cellular component in buccal swab consists of buccal epithelial cells, with a minor presence of immune cells (25). Therefore, the majority of buccal swab samples displayed the recipient’s pre-HSCT HLA genotype. Saliva contains a mixture of epithelial cells shed from the buccal mucosa and immune cells secreted by salivary glands or those that have migrated locally or systemically (26, 27). This could explain the mixed HLA genotype observed in saliva samples, reflecting contributions from both the donor and the recipient. While this conclusion has been widely acknowledged, our findings offer robust evidence to support this well-established notion, thereby providing valuable insights for the judicious selection of samples in HLA genotyping for patients who have previously undergone allogeneic HSCT.

Additionally, we reported several unexpected findings rarely documented in prior studies. Specifically, donor-specific HLA gene chimerism and LOH were detected in certain buccal swab and saliva samples. More strikingly, one recipient’s buccal swab exhibited a complete donor HLA genotype two and a half years post haplo-HSCT. Although we did repeated experiments on a newly collected buccal swab sample from this patient, the possibility of sample contamination, blood cell admixture, or technical bias during the DNA extraction process could not be entirely ruled out as potential causes for this unexpected result. These findings we believe may prompt further consideration and discussion among peers, fostering continued exploration in this area.

It is important to note that the cellular composition of buccal and saliva samples in our study is not controlled. To address this limitation, the utilization of immunomagnetic depletion of human CD45^+^ cells may be a promising strategy for future investigation. Should this method successfully achieve adequate cell enrichment for DNA extraction, it would facilitate the isolation of two distinct fractions: a negative selection (CD45^-^ fraction) and a positive selection (CD45^+^ fraction). The CD45^-^ fraction, predominantly composed of salivary or epithelial cells, would offer a purified cell population that enables more accurate and targeted analyses of non-hematopoietic components. Concurrently, the CD45^+^ fraction comprising hematopoietic cells could serve as an invaluable comparative control for HLA genotyping within the peripheral blood compartment.

Stable mixed chimerism is usually not associated with poor outcomes in non-malignant diseases such as aplastic anemia when donor-derived progenitor cells produce a sufficient number of erythrocytes (8). However, recipient chimerism in patients with hematologic malignancies may foretell disease relapse. In our results, two peripheral blood samples, four buccal swabs and four saliva samples presented a donor-recipient chimeric state of HLA genotype. Hematopoietic stem cells belong to early undifferentiated cells and have the potential to differentiate into epithelial cells of the liver, lung, gastrointestinal tract, and skin (28). Tran SD et al. reported that donor-derived hematopoietic stem cells were capable to migrate into the recipient’s cheek and differentiate into buccal epithelial cells *in vivo* (29). These reports may explain our results that one buccal swab exhibited the donor’s HLA genotype and four buccal swabs showed chimerism status of the recipient and donor. However, to accurately trace the cellular origin, future studies could integrate genome-wide profiling techniques, such as bisulfite sequencing or array-based approaches. These methods would enable us to verify whether the observed epigenetic signatures are indicative of the recipient’s native salivary or buccal epithelial cells or, alternatively, if they have transdifferentiated from the donor’s hematopoietic stem cells. Consistently, numerous studies have reported that the STR results of the buccal and gastrointestinal epithelial cells exhibited the chimerism of the recipient and donor after allogeneic HSCT (30). In future, the *in vivo* tracking approach with a mouse model may also be employed to confirm the presence of donor derived cells as described previously (31). The hematopoietic stem cells extracted from the donor are labeled with a membrane-bound fluorescent dye PKH26 and the spatiotemporal distribution of donor-derived cells can be systematically tracked using flow cytometry or fluorescence microscope at different time points following transplantation.

Genomic LOH through acquired uniparental disomy (aUPD) occurs when the region encompassing the HLA genes on chromosome 6p is lost in leukemic cells and the remaining chromosome is duplicated by the cellular machinery. LOH can occur in pretransplant blood samples from patients with a high number of leukemic blasts, which always necessitates re-typing with buccal swab DNA (32, 33). HLA loss in leukemia is more prevalent at disease relapse and especially in relapsing cells after HLA-mismatched HSCT (17, 34). Loss of the mismatched recipient-specific HLA haplotype allows immune escape from donor T cells (15). Differently, in the studied patient diagnosed with AML, LOH was also observed in both the buccal swab and saliva samples 28 months after haplo-HSCT. Consistent with our results, LOH at the HLA loci has been also detected in the buccal swab sample of a patient with leukemia who received haplo-HSCT (35). LOH observed in buccal swab and saliva might be caused by the migration and differentiation of hematopoietic stem cells with LOH, but more intensive studies are required to verify this hypothesis. Additionally, although the peripheral blood of this AML patient exhibited the donor’s HLA genotypes, the two haplotypes were in imbalance, with the donor-recipient matched genes occupying a higher proportion than the mismatched genes. This might be caused by the coexistence of donor-derived blood cells and the leukemic cells with LOH. To validate this assumption, future research should consider integrating disease-burden metrics, such as measurable residual disease (MRD) detection through NGS, and establish correlations between these metrics and the degree of HLA allelic imbalance observed in the peripheral blood compartment via NGS.

In conclusion, our findings demonstrate that HLA genotypes in different tissues of post-haplo-HSCT patients are variably influenced by donor hematopoietic stem cell engraftment. Therefore, for HLA matching assessments in patients undergoing second HSCT, multi-tissue sampling should be prioritized to enable more comprehensive genotyping, which facilitates more informed clinical decision-making and optimized therapeutic strategies to improve patient survival. Additionally, the chimeric patterns and complete donor’s HLA genotype observed in the recipient’s buccal swab warrant further discussion among clinicians and researchers, encouraging continued investigation into the long-term genetic effects of allogeneic transplantation.

## Data Availability

The original contributions presented in the study are included in the article/**Supplementary Material**. Further inquiries can be directed to the corresponding authors.

## Supplementary material

Supplementary Table S1 The HLA genotyping results across different tissues of all the investigated subjects.
